# Multiple Export Mechanisms for mRNAs

**DOI:** 10.3390/cells4030452

**Published:** 2015-08-28

**Authors:** Mildred Delaleau, Katherine L. B. Borden

**Affiliations:** Department of Pathology and Cell Biology, Institute for Research in Immunology and Cancer, Université de Montréal, Montréal, QC, H3C 3J7, Canada; E-Mail: mildred.delaleau@umontreal.ca

**Keywords:** nuclear mRNA export, selective export, NXF1 pathway, CRM1 pathway

## Abstract

Nuclear mRNA export plays an important role in gene expression. We describe the mechanisms of mRNA export including the importance of mRNP assembly, docking with the nuclear basket of the nuclear pore complex (NPC), transit through the central channel of the NPC and cytoplasmic release. We describe multiple mechanisms of mRNA export including NXF1 and CRM1 mediated pathways. Selective groups of mRNAs can be preferentially transported in order to respond to cellular stimuli. RNAs can be selected based on the presence of specific cis-acting RNA elements and binding of specific adaptor proteins. The role that dysregulation of this process plays in human disease is also discussed.

## 1. Introduction

Accurate and dynamic control of gene expression is critical for all cells in order to respond to cellular stresses, environmental stimuli and to properly regulate proliferation and growth. Gene expression can be controlled not only at the levels of transcription and translation, but also by controlling the nuclear-cytoplasmic export of mRNAs that encode proteins involved in these cellular processes. Specific subsets of mRNAs can be differentially exported leading to alterations in the proteome by controlling the levels of transcripts available to the translation machinery. This specificity is often underpinned by the presence of specific sequence elements within the untranslated regions (UTRs) of these transcripts. These elements act as USER codes allowing groups of transcripts acting in similar activities to be coordinately exported as in the RNA regulon model [1,2].

**Figure 1:**
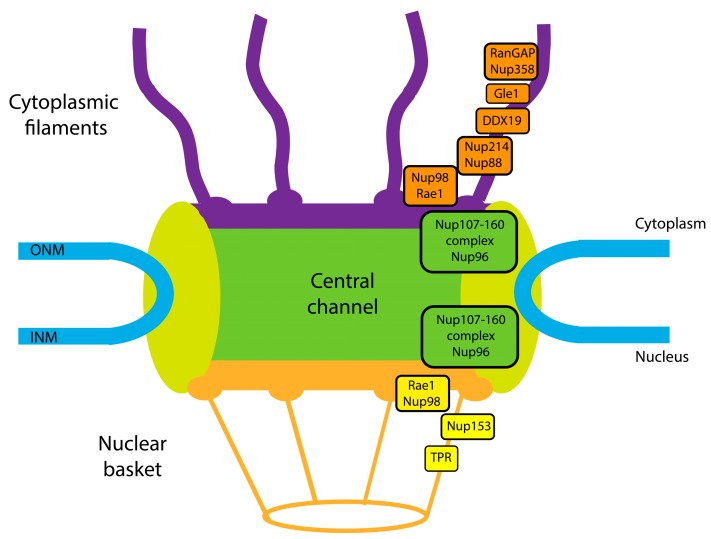
Schematic cross-section of the nuclear pore complex showing a subset of Nups discussed in this review as well as the major features of nuclear pore complex (NPC). INM indicated inner nuclear membrane and ONM, outer nuclear membrane.

To be efficiently exported transcripts must undergo several maturation steps including capping, splicing and 3′ end formation. Then transcripts must traverse the nuclear envelope generally via the nuclear pore complex. This involves docking onto the nuclear basket, transiting the central channel of the nuclear pore and being released from the cytoplasmic fibrils (Figure 1) [3,4,5,6,7]. mRNAs bound to particular protein co-factors associate with specific transporters to traverse the nuclear pore complex (NPC). Once released into the cytoplasm, mRNAs can then become available to the translation machinery. All of these steps are highly regulated and show differential specificity and dynamic response to a wide variety of stimuli. Nuclear mRNA export was once considered to be a constitutive housekeeping activity but now is seen as a highly regulated process that can be dysregulated in and contribute to human disease. In this review, we highlight some of the recent advances in our understanding of mRNA export in mammals and discuss their implications for human disease.

## 2. General Features of Bulk mRNA Export

**Figure 2:**
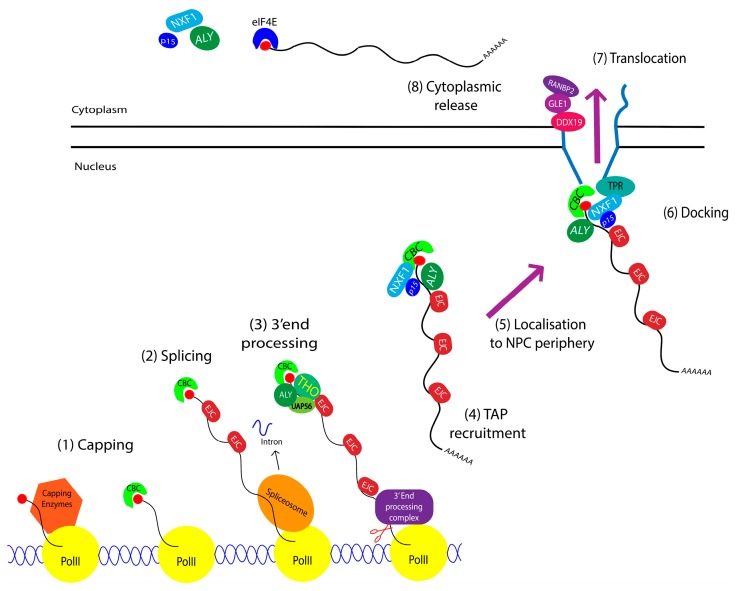
Global overview of processing and export. The different processing steps include methyl 7-guanosine capping (1); splicing (2) and the 3′ end processing (3); The mRNA is then considered mature and can be exported. NXF1 is the transporter for bulk mRNA and works in association with p15 (Nxt1) (4); Next, the export mRNP, reaches the nuclear pore complex (5); where NXF1 interacts with TPR (Translocated promoter region) to dock mRNP to the nuclear basket (6); The interaction with TPR will initiate the translocation through the nuclear channel (7); Interaction with the cytoplasmic fibrils of the NPC (composed of RANBP2) and key co-factors (Gle1 and DDX19) will release the mRNA into the cytoplasm (8).

Transcripts in the nucleus are transcribed as pre-mRNA and after multiple processing steps [8], these now mature mRNAs are transported to the cytoplasm to allow their translation into proteins (Figure 2) [6,7,9,10,11,12]. Recruitment of export factors to nascent mRNA start co-transcriptionally [13,14] and correctly processed mRNAs are targeted for export in the form of large ribonucleoprotein complexes called mRNPs [15]. The processing of pre-mRNA to mature mRNA includes three key steps: capping, splicing and 3′ end processing. The mature mRNA is then targeted to and translocated through the nuclear pore complex (NPC) to reach the cytoplasm and can then be translated [3,6,9,16,17]. The most common transporters for mRNA export are the protein NXF1 (also known as TAP) [18,19,20] and CRM1 [3,4,5,21]. Both NXF1 and CRM1 utilize adaptor proteins to increase the affinity of NXF1 for its RNA targets [22] or in the case of CRM1, to associate with the target RNA [4,5,23].

The typical means to transverse the nuclear envelope is through the nuclear pore complex (NPC). NPCs permit the passage of proteins, RNAs and solutes, between the nucleus and the cytoplasm. NPCs are 60–125 MDa structures composed of approximately 30 different proteins called nucleoporins (Nups) [24,25,26]. The Nups form an octagonal structure containing a central cylindrical channel, cytoplasmic fibrils, and a nuclear basket. The Nups can be divided into 3 different groups: membrane Nups that anchor NPCs to the nuclear envelope, structural Nups (also called scaffolding Nups) and the FG Nups which contain repetitive sequence of phenylalanine and glycine called FG repeats and typically play a role in the transit of cargoes through the central channel [24,25,26,27,28,29,30]. While nucleocytoplasmic transport of small molecules (less than about 40 kDa) occurs via diffusion [31]; larger complexes including mRNA export RNPs need energy to facilitate directionality [32,33,34,35]. For bulk mRNA, this is achieved through an ATP helicase cycle involving Gle1, DDX19 and inositol hexakisphosphate (IP6) [32,34,36,37,38,39]. Once export RNPs arrive at the nuclear pore, export can be generally considered as three distinct steps: docking onto the nuclear basket, transit through the central channel and release of mRNA cargoes at the cytoplasmic fibrils [3,6,7].

## 3. NXF1 Mediates Export for Bulk mRNA Export

NXF1 mediates the nuclear export of bulk mRNAs [3,6,9,16]. NXF1 associates with key factors including REF/ALY and proteins of the THO complex in order to directly bind target mRNAs with high affinity [19,22,40]. Association of the mature mRNP with NXF1 allows docking with the nuclear basket nucleoporins including TPR, Nup153, Nup98 and Rae1, and subsequent translocation through the central channel [41,42,43].

The critical role for NXF1 in the pathway is highlighted by the observation that NXF1 is essential for cell viability and NXF1 knockdown leads to accumulation of bulk poly(A) mRNA in the nucleus [19,44,45,46,47]. NXF1 was first identified as an export factor in Mason Pfizer monkey virus RNA containing the constitutive transport element (CTE) [48,49,50], and later studies showed it was important for bulk cellular mRNA export [47,50]. NXF1 is composed of multiple domains [6,18,22,42,51,52,53,54,55] including: the RNA recognition motif (RRM) that permits NXF1 to bind non-specific RNA with a low affinity but with increased affinity in the presence of adaptors such as ALY/REF; and a leucine rich repeat domain (LRR) in conjunction with the RRM domains can bind the CTE element. The remaining two domains of NXF1 permit the translocation through the central channel by interacting with the FG Nups.

For NXF1 mediated bulk mRNA export, the most common adaptor is ALY/REF [22,40]. In order to associate with ALY/REF, NXF1 must associate with the transcription export complex (TREX) [56,57,58,59]. Capping and splicing events permit the recruitment of the TREX to the 5′ end of the mRNA [60]. The TREX complex is composed of the THO complex (Thoc1/hHpr1, Thoc2, Thoc3/hTEX1, Thoc5/FMIP, Thoc6 and Thoc7) [61] and the proteins UAP56, ALY/REF and CIP29. The THO complex assembles in an ATP dependent manner with the RNA helicase UAP56, ALY/REF and CIP29 [62,63]. The ability of these factors to associate with the export RNP is reliant on correct mRNA processing thereby providing a link between mRNA processing and export [64]. For instance, UAP56 is also a component of the exon junction complex (EJC) [65], which is deposited on the mRNA as a marker of completed splicing (see below). Another example is that the THO complex with ALY/REF interacts with the nuclear Cap Binding Complex (CBC) [66], which binds the m^7^G cap on the 5′ end of the mRNA. Thus, capping is also linked to more efficient mRNA export. The THO complex was shown to interact with mRNA during 3′ end formation, and mutation of THO leads to nuclear accumulation of RNP containing nuclear pore components and polyadenylation factors [67]. Thus, generally, TREX recruits NXF1/p15 to cargo mRNAs once they have been fully processed thereby favouring the export of mature transcripts.

After processing of mRNAs, an export-competent NXF1-mRNP is formed in the nucleoplasm which needs to reach the nuclear periphery in order to dock onto the nuclear basket and be transported. One possibility is that another complex, TREX-2, transports the mRNP from the place of transcription and mRNA processing to the nuclear basket [68,69]. In humans, TREX-2 is comprised of GANP, ENY2, CETN2/CETN3, PCID2 and DSS1. GANP is generally considered to be the scaffold of the TREX-2 complex and interacts directly with NXF1 [70]. Through TREX-2, the export mRNP can associate with the TPR protein at the nuclear basket of the NPC [71]. It is not clear whether GANP is a general factor or facilitates export of a subset of specific transcripts [72]. In some studies, transcripts that were GANP dependent were transported about two times more quickly than those that were not [72]. This raises the possibility that GANP mediates a fast track export route for transcripts that encode proteins involved in specific functions presumably to facilitate a rapid adaptation to changes in cellular environment.

After the docking of the mRNP to the nuclear basket, the translocation proceeds via interactions with FG Nups along the central channel to translocate through the NPC [41,42,43]. Nup98 and Rae1, which are localized at the beginning of the central channel are key [42,73]. It was proposed that Rae1 may act to deliver NXF1 to Nup98 and thus be the first in a series of interactions with the Nups [73]. NXF1 associates with Nup62 inside the central channel [42]. The mechanism of mRNP translocation through the nuclear channel is still under discussion, to date several nuclear transport models have been proposed [74,75,76,77,78,79,80,81]. The time frame of export is rapid. Early studies using β-actin mRNA with a yellow fluorescent protein fused to the MS2 protein tag found that these transcripts were exported in the 180 ms timeframe where docking was on the order of 80 ms, transit through the central channel 5–20 ms and release 80 ms [82]. Several studies show that docking at nuclear basket does not mean that export will be successful. One report estimated that approximately 25% of the mRNPs that docked to the nuclear basket were actually exported, the remaining 75% returning to the nucleus [83]. These studies estimated the complete export process to take on the order of 65 ms to several seconds [83]. Consistently, other groups reported that 35% of docking events resulted in successful transport [84]. Using high temporal resolution, SPEED microscopy studies suggested export was on the order of 20 ms and docking of the mRNP with the NPC on the order of 10 ms [85]. The range of export times reported likely arises due to the different experimental systems employed to measure these processes. Taken together, it is clear that export is a rapid process with lower than expected success rates.

Upon exit from the central channel on the cytoplasmic side, mRNAs associate with the cytoplasmic fibrils of the NPC, and the mRNA cargoes must be released into the cytoplasm followed by recycling of export factors back to the nucleus. This process is highly regulated and impacts on the efficiency of mRNA export. The long fibrils of the cytoplasmic face are mainly comprised of Nup358/RanBP2 [86]. This factor contains binding sites for NXF1, RanGAP, Ran and others [86,87]. It associates with the NPC via Nup88 and Nup214 [88]. RanBP2 knockout mice have severely impaired mRNA export while RanBP2 hypomorph mice do not have impaired bulk export but do have elevated export of specific mRNAs [89,90]. Interestingly, RanBP2 hypomorph mice develop spontaneous tumours [39,91]. For bulk mRNA export, cargoes are released in an ATP dependent manner via the DEAD box helicase DDX19 and Gle1 [32]. This release step depends on the binding of the signalling molecule inositol hexakisphosphate (IP6) to Gle1 and the corresponding complex stimulates DDX19 binding to the mRNA cargo and triggers ATP hydrolysis and transcript release [3,33,34,36,39]. In the cytoplasm, the mRNP is remodelled, for example the CBC complex is replaced by eIF4E thereby permitting steady-state translation [92].

## 4. The NXF1 Pathway and Export of Specific Transcripts

**Figure 3:**
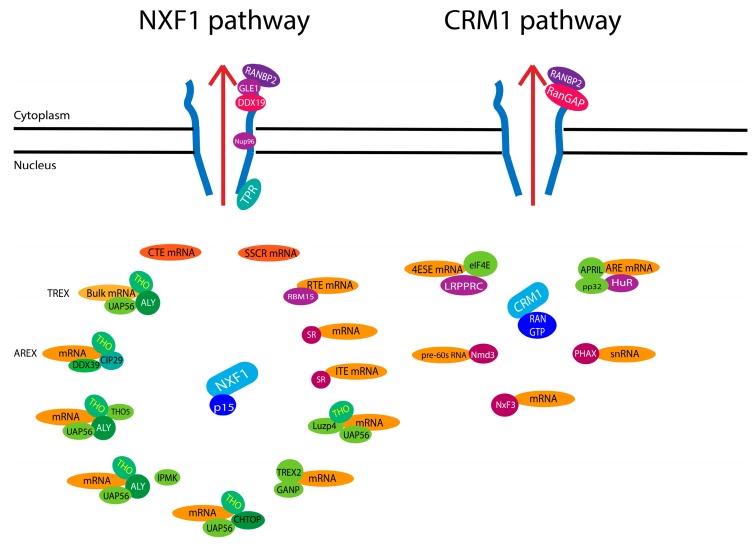
NXF1 and CRM1 export show plasticity by interacting with different adaptor proteins as described in the text. NXF1 will interact with different adaptors or with the RNA directly as with constitutive transport element (CTE) or signal sequence coding region (SSCR) containing mRNAs. This mRNP docks on the NPC via TPR and is translocated through the central channel. At the cytoplasmic fibrils, bulk mRNA is released into the cytoplasm by the action of RANBP2, DDX19 and Gle1. CRM1 also interacts with different adaptors in presence of Ran-GTP, and translocates through the NPC to reach the cytoplasmic fibril. Here the complex is dissociated by RanBP2 and RanGAP which hydrolyses Ran-GTP to Ran-GDP and permits the release of the mRNA into the cytoplasm.

Above we discuss a generalized model for bulk mRNA export. Importantly, by altering the composition of the export RNP, specificity can be introduced into the system (Figure 3) [93,94]. In the case of the NXF1 export pathway, NXF1 can use different protein components to enable selection of specific mRNA cargoes. For instance, different proteins from TREX can mediate specific export. The DEAD box helicase DDX39, which is highly homologous to UAP56, binds ALY/REF in the TREX1 complex [58,95,96]. NXF1 can also form another specific mRNA export complex known as AREX for alternative mRNA export involving CIP29 and DDX39. AREX is used to export of a subset of mRNAs including some involved in mitosis [97]. The depletion of DDX39 leads to chromosome arm resolution defects and failure of cytokinesis by acting on Survivin and PRC1 mRNA export [97].

Another factor that can act as an adaptor for NXF1 is the TREX component THOC5 [98]. This protein is not necessary for the nuclear export of bulk mRNAs but is necessary for the export of the HSP70 mRNP where THOC5 is able to bind NXF1 in association with ALY/REF to expedite export of these mRNAs [99]. THOC2 and THOC5 regulate stem cell pluripotency and somatic cell differentiation by influencing the nuclear mRNA export of the pluripotency encoding factors Nanog, Sox2, Klf4 and Esrrb [100]. In fact, nuclear retention of these transcripts was observed after inhibition of THOC2 or THOC5. The knockdown of THOC5 reduces the interaction between these mRNAs and THOC2. The expression of THOC5 is reduced during differentiation, which permits selective mRNA export leading to decreased expression of the pluripotency factors during differentiation [100]. Another TREX component is ChTOP competes with ALY/REF for NXF1/p15 and can potentially modulate the export of a subset of targeted transcripts [101].

The serine-arginine rich (SR) proteins also form specific NXF1 dependent export mRNPs [102]. Three different SR proteins SRp20, 9G8 and ASF/SF2 can compete for ALY/REF binding in the complex. Initially, 9G8 and SRp20 were shown to recognize a 22 nucleotide motif in the histone H2A mRNA denoted the Intronless Transport Element (ITE) [102]. Both of these factors bind this element and recruit TAP thereby promoting export. Since these initial findings, SR proteins have been shown to also function in the export of some spliced transcripts. Here, the phosphorylation state of the SR proteins plays an important role. For instance, the hyperphosphorylated 9G8 is recruited to pre-mRNA prior to splicing, becoming hypophosphorylated after the splicing, which permits recruitment of NXF1 [103,104]. In the cytoplasm, rephosphorylation of the SR proteins releases NXF1 and the mRNA cargo. This demonstrates not only that there are multiple NXF1 complexes which can act in specific mRNA export but also provides an important example of how signalling modulates mRNA export of these specific transcripts.

Specificity in terms of forming complexes for specific transcripts often requires defined sequence elements in these mRNA cargoes. This provides binding specificity by recruiting certain NXF1 partner proteins. For example, RBM15, is implicated in the export of mRNAs containing the RNA transport element (RTE) [105]. RTE was first identified in mouse transposons and is highly similar to the constitutive transport element of type D retroviruses. The RTE minimal element contains 4 internal stem loops which interact with RBM15 [106]. RBM15 also facilitates the recognition of NXF1-mRNP complexes by DDX19 during translocation of general mRNA [107].

Another example of NXF1 dependent selective mRNA export was described for mRNAs encoding proteins involved in genome duplication and repair, e.g., RAD51, CHEK1 and FANCD2 [108]. The interaction of ALY/REF with these mRNAs is controlled by the enzyme inositol polyphosphate multikinase (IPMK). IPMK is a kinase involved in synthesis of inositol phosphates and phosphoinositide turnover. The recognition of RAD51 transcripts by ALY/REF required IPMK [108]. The IPMK product restores the interaction between RAD51 and ALY/REF in IPMK-depleted cell extracts. A sequence motif in the 3′ UTR of RAD51 acts as the recognition motif for ALY/REF. This study suggests that IPMK protein regulates, at least in some cases, the sequence-based selection of transcript for export via ALY/REF [108].

Recent reports suggest that in cancer cells the Luzp4 protein, which associates with UAP56 and NXF1, is overexpressed suggesting dysregulated mRNA export here [109]. Indeed, Luzp4 complements ALY/REF knockdown *in vivo* demonstrating its functional role in this process [109]. The subsets of transcripts that are selectively exported with Luzp4 are undetermined, however it seems likely that these factors are required for efficient growth of melanoma cells.

Importantly, proteins within the NPC can also modulate specificity of mRNA export. For instance, Nup96, a constituent of the Nup107-Nup160 complex, plays a role in the export of mRNAs coding for cell cycle regulators and immune response factors e.g., MHCI, MHCII, β2 microglobulin and CDK6 [110,111]. The precise mechanism for this Nup96 activity is still not known but it can act at the NPC or in the nucleoplasm due to the presence of a nucleoplasmic population of this Nup [93,112,113].

In some cases, NXF1 is able to export transcripts by direct interaction with RNA elements such as the CTE described above [18]. In mammalian cells, NXF1 also recognizes the signal sequence coding region (SSCR) which is found on transcripts coding for secretory proteins and target them for translocation across the membrane of the endoplasmic reticulum [114]. SSCR is also an export USER code for mRNAs lacking introns or functional caps [115]. This SSCR-mediated export pathway requires NXF1 but is not TREX-dependent [115].

## 5. CRM1 and Specific mRNA Export

The above examples have focussed on NXF1. However, not all transcripts require the NXF1 receptor to associate with the NPC. Indeed, subsets of mRNAs use the karyopherin CRM1 (Figure 3). Classically, CRM1 plays a major role in the protein export of proteins with leucine-rich nuclear export signals (NES) [116,117]. Importantly, CRM1 directly interacts with the NPC [118]. To date, CRM1 does not bind RNA itself, but through association with NES containing protein co-factors and using this strategy plays an important role in the export of small nuclear RNAs (UsnRNAs), ribosomal RNAs and a specific subset of mature mRNAs [5,117,119]. CRM1 binds its cargoes with high affinity only in the presence of Ran-GTP. In the cytoplasm, cargo release requires the Ran GTPase activating protein (RanGAP) and either RanBP2 (the cytoplasmic fibril) or the small soluble RanBP1 enabling GTP hydrolysis of Ran. This allows CRM1 cargo release. Similar to NXF1 mediated export, Nup88, Nup214 and RanBP2 play critical roles in the release and recycling of cargoes [5].

Specific factors in the RNP again permit selection of specific mRNAs. One such adaptor is HuR (Human antigen R). HuR plays a wide variety of roles in RNA processing within cells through its association with the AU rich element (ARE) in the 3′ UTR of transcripts [120]. These elements are well known to play roles in mRNA stability [120]. However, CRM1 associates with pp32, APRIL and HuR to export a subset of ARE containing mRNAs [121]. The CRM1 dependence is demonstrated by treatment with the CRM1 inhibitor Leptomycin B, which leads to accumulation of some ARE containing transcripts but not bulk mRNA [122,123]. Not all transcripts that rely on CRM1 for export are dependent on HuR. For instance, the ARE containing transcript human interferon alpha is transported by CRM1 but is HuR independent [124,125]. Further plasticity is suggested by the observation that CRM1 acts in NXF3 mediated export of certain transcripts in specific tissue contexts [126]. NXF3 is a NXF1 family member but does not bind to Nups and thus uses CRM1 for translocation through the NPC. The specific sequence elements required for this export are currently unknown [126].

CRM1 plays a critical role in eIF4E-dependent mRNA export. Traditionally, eIF4E is known to act in cap-dependent translation. However up to 70% of eIF4E is found in the nucleus and here it functions in the export of a specific subset of mRNAs [94,127,128,129,130]. Target mRNAs contain in their 3′ UTR a secondary structure element of approximately 50 nucleotides which is called the 4E-sensitivity element (4ESE) [94]. The transcript must be capped and contain the 4ESE to be an eIF4E export target [94,128]. Currently, it does not appear that eIF4E directly recognizes the 4ESE element but rather this occurs through the export adaptor LRPPRC likely through a pentatricopepetide repeat domain [131]. LRPPRPC directly binds both the 4ESE element and the eIF4E protein [131]. LRPPRC binds (directly or indirectly) to CRM1 allowing transit through the NPC. The reliance on CRM1 is demonstrated by the inhibition of eIF4E dependent mRNA export by leptomycin B and the observation that knockdown of NXF1 did not affect this pathway [94]. To date, RIP-Seq experiments indicate that eIF4E associates with approximately 2300 transcripts in the nuclear fraction indicating that it can modulate the export of a wide variety of transcripts [132]. Interestingly, some protein co-factors in this pathway are common to both NXF1 and the eIF4E/CRM1 pathway including UAP56, hnRNPA1 and DDX3 [131]. However, NXF1, REF/ALY and CBC are not part of the eIF4E dependent mRNA export pathway [131]. Endogenous 4ESE containing mRNAs are targets of both the bulk and eIF4E dependent export pathways with 3′ UTRs of thousands of nucleotides in length and contain many competing USER codes [94,128,129]. Thus, eIF4E competes with bulk pathway to enhance export of specific transcripts.

Interestingly, eIF4E can modulate the composition of the NPC itself, in order to expedite the export of its target transcripts [90]. Specifically, eIF4E overexpression leads to dramatically reduced RanBP2 levels and alterations in the localization of Nup214 [90]. Conversely, eIF4E increases the mRNA export of RanBP1 transcripts which presumably increases the efficiency of release and recycling of eIF4E-CRM-exRNPs on the cytoplasmic face of the NPC. RanBP2 reduction was sufficient to increase eIF4E dependent but not bulk mRNA export while RanBP2 overexpression impaired eIF4E dependent mRNA export as well as its oncogenic potential. This is consistent with the observation that RanBP2 hypomorph mice get cancers more readily than littermate controls [91]. Importantly, RanBP2 hypomorphs have multiple defects which likely also contribute to the observed phenotype [91]. These findings suggest that RanBP2 slows down release and recycling of eIF4E dependent mRNA export cargoes [90]. Thus, to enhance export, eIF4E suppresses RanBP2 to reduce sequestration. eIF4E is highly elevated in a wide variety of human cancers [127] suggesting that eIF4E can reprogram the NPC to promote proliferation and survival.

## 6. Other Exits for mRNA

**Figure 4:**
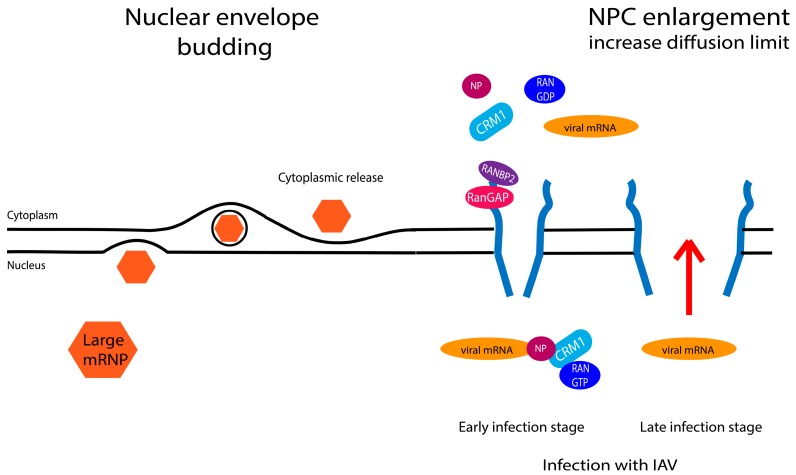
Schematic presentation of different exits routes for mRNA. On the left, the nuclear budding for large mRNP, and on the right is a schematic representation of the NPC enlargement induced by the Influenza A virus (IAV) infection.

Recently, it was shown that mRNPs that are too large to translocate through the center channel could bypass the NPC by using a mechanism similar to the Herpes virus nuclear egress [133,134,135,136,137] (Figure 4). This process is termed nuclear envelope budding. It was shown that during synapse development, large mRNP granules exit the nucleus by budding through the nuclear envelope (NE). This budding involves phosphorylation of the nuclear lamin by an atypical protein kinase C [135]. The phosphorylated lamin permits the invagination of the inner nuclear membrane (INM) into the NE lumen. Then a vesicular fusion with the outer nuclear membrane (ONM) permits the release into the cytoplasm. This process permits the export of large mRNPs without remodeling of the NPC [135].

Another interesting RNP export modality was discovered while investigating mRNA export in Influenza A viruses (IAV) [138]. Electron microscopy studies show that IAV enlarges the nuclear pores in infected cells by around 20 nm (a final size of around 50 nm) [139,140] (Figure 4). The increase in pore diameter facilitates the translocation of large protein complexes. This widening is due to a virus-induced cellular caspase activity. Newly synthetized viral RNPs normally use the CRM1 transporter with the viral structural nucleoprotein NP as an adaptor. However the enlargement of the nuclear pore seems to dramatically change the diffusion limits of NPCs at late infection stages, since proteins of ~125 kDa are able to accumulate in the cytoplasm via passive diffusion [140]. This allows the passive diffusion of viral RNPs which can complement CRM1-dependent RNP export mechanisms to increase the production of infectious virus progeny at late stages of the replication cycle [139]. Future studies will determine whether this mechanism is also relevant to host cells.

## 7. RNA Processing and Modification Can Modulate RNA Export

Capping, splicing and poly (A) tail formation are all known to increase efficiency of mRNA export (see above). Each step is marked by the deposition of specific factors which better allow transport factors to associate with the RNA [6,8]. However, there are ways around these processing events. For instance, while splicing is important for export, intron-retained RNAs can be exported [49,141]. Normally, export of unspliced or intron-retained RNAs is blocked at the nuclear basket by TPR. Indeed, the inactivation of TPR leads to an increase in export of intron-retained mRNAs [142]. Export of intron-retained transcripts leads to translation leading to proteins with different functionalities. Classically, viruses export unspliced RNAs for packaging into virions, e.g., the RRE (Rev response element) in HIV binds the viral protein REV which is then exported by CRM1 by virtue of its NES [143]. However, human mRNAs can also be exported with introns. For example, intron retained forms of NXF1 mRNA itself are exported by the NXF1 protein [49,141].

Another question that arises given the relevance of splicing to export is how transcripts without introns are efficiently exported given they do not associate with the EJC. Interestingly there have been multiple export pathways which allow export of such transcripts. For instance, many intronless mRNAs contain a specific element in the 3′ UTR known as cytoplasmic accumulation regions (CAR) [144]. The TREX complex interacts with this element [145] via splicing factors PRP19 and U2AF65 [144]. In this way, naturally intronless mRNA bypass splicing dependent export by recruiting the mRNA export machinery via the CAR element [144]. The histone mRNAs provide another example for a mechanism of intronless mRNA export. Histone mRNAs not only lack introns but are also not poly-adenylated [146]. They undergo a cleavage reaction that ends the mRNA with a 3′ conserved stem-loop (SL) [146]. The SL structure is recognized by the SL binding protein (SLBP), which functions in their export. In fact the knockdown of SLBP decreases export and leads to an accumulation of processed histone mRNA in the nucleus [147]. However, some histones, e.g., H2B mRNA, are exported via the SR proteins as described above.

Finally recent studies indicate that there are other covalent modifications can modulates RNA fate. For example, N6-methylation of RNAs can modulate mRNA export, as well as translation efficiency and stability. This process is reversible suggesting that even fully matured mRNAs could have their export modulated by these modifications [148].

## 8. RNA Export and Disease

During the last decade, the dysregulation of mRNA export has been implicated in numerous diseases [23,149,150]. Dysregulation of different pathways can lead to different effects. For instance osteogenesis imperfecta type I occurs when there is a mutation on collagen pre-mRNA sequence causing improper splicing which results in nuclear sequestration of the transcripts and subsequent diminution in collagen levels [151]. Mutation on the pre-mRNA is also the cause of the Myotonic dystrophy type I [152]. In this case, an abnormal CGU expansion in the 3′ UTR of the DM protein kinase mRNA results in transcripts that are resistant to export due to a defect in their processing [153,154]. In fact, the expanded CGU repeats sequester the mRNA-splicing factor MBNL1 leading to mis-splicing of essential MBNL1 regulated mRNAs [155]. Another mechanism that can cause retention of mRNA in the nucleus is mutation in the export or processing factors themselves. For example, the mutation of the export factor GLE1 is linked to two motor neuron diseases: Lethal congenital contracture syndrome-1 and Lethal arthrogryposis with anterior horn cell disease [156]. Here, mutation of GLE1 results in a splicing error causing the insertion of 3 amino acids in the protein sequence that alters GLE1 structure and thus its export release activity [35,156,157].

Dysregulation of mRNA export is also observed in many type of cancers [4]. The export factor CRM1 is over-expressed in several different human tumors e.g., gliomas, cervical cancer and pancreatic cancer [158,159]. The elevated levels of CRM1 cause an increase in the mRNA export of subset of transcripts that contribute to proliferation and survival, and also of its protein export function in cervical cancer, for example [160]. Other export factors can also be elevated in cancer e.g., THO, ALY/REF and GANP [161,162,163]. Another example of dysregulation of mRNA export is due to the modulation of the NPC by a changing levels of specific Nups or through chromosomal translocations leading to Nup fusion proteins [150]. Another example is Nup88 elevation is found in several malignancies and correlated with higher tumour grade [164,165]. On the other hand, downregulation of Nup96 is implicated in decreased export of specific mRNAs, which leads to accelerated cell cycle progression [110,111]. Reprogramming of the NPC was observed during viral infection with the vesicular stomatitis virus (VSV) where the virus disrupt the interactions between Nup98 and Rae1 to inhibit host cell mRNA export [166]. Heterogeneity of the NPC in three human cancer cell lines has also been observed [167]. This could also impact mRNA export as well. Another example of NPC programming and elevated mRNA export contributing to cancer is provided by eIF4E as described above.

## 9. Conclusions and Perspectives

Export of mRNAs is often a selective and highly regulated process that can impact on cell physiology and disease. As described above, groups of RNAs can be elegantly selected by virtue of their cis-acting RNA elements also known as USER codes and trans-acting factors. Historically, mRNA export was considered a simple conduit between transcription and translation. However, mRNA export critically controls the availability of transcripts to the translation machinery and thus profoundly affects the proteome and the response of cells to wide variety of stimuli.

There are many open questions and exciting future directions for the field. For instance, the extent that signaling pathways impact on the export process not only at the level of modification of relevant proteins, but also on modification to the transcripts themselves e.g., methylation [168]. It will be interesting to understand the extent to which tissue specific differences in the NPC affect mRNA export (such as observed in human cancer cells [167]) and further, the variety of means by which mRNA export can modulate the NPC to promote specific export such as the case for eIF4E [90]. A deeper understanding of the RNA export elements *i.e.*, USER codes for export is important. Currently there is much more known about the precise elements that modulate virus export than host cell export. Such studies are important for understanding how groups of mRNAs could be dysregulated leading to cellular reprogramming and how such events could contribute to human disease.
